# Cohort profile: Famenin Brucellosis Cohort Study

**Published:** 2019-08-03

**Authors:** Fariba Keramat, Manoochehr Karami, Mohammad Yousef Alikhani, Saeed Bashirian, Abbas Moghimbeigi, Maryam Adabi

**Affiliations:** ^1^Brucellosis Research Center, Hamadan University of Medical Sciences, Hamadan, Iran; ^2^Research Center for Health Sciences, Hamadan University of Medical Sciences, Hamadan, Iran; ^3^Department of Epidemiology, School of Public Health, Hamadan University of Medical; ^4^Social Determinants of Health Research Center, Hamadan University of Medical Sciences, Hamadan, Iran; ^5^Modeling of Noncommunicable Diseases Research Center, Hamadan University of Medical Sciences, Hamadan, Iran

**Keywords:** Cohort Study, Brucellosis, Serologic Tests, Iran

## Abstract

**Background:** To achieve preventive and controlling activities of Brucellosis, we aimed this study as the first prospective cohort survey on brucellosis in Iran. This cohort in different phases from 2016 until 2020 going to investigate about brucella infection in the selected population of Famenin, a city located in Hamadan province, west of Iran.

**Study design:** A prospective cohort study.

**Methods:** At the first phase of the study, Famenin inhabitants including urban and rural people were studied from September to December in 2016. All identified household’s people referred to specified health centers and clinically visited. Blood sampling was done, then these subjects were joined and the follow-up was initiated. At the next step, the blood samples were examined using Wright kits and 2ME test for diagnosis the seroprevalence of brucellosis. Participants will be followed up for next years to examine clinical profiles of brucellosis and complete investigation about the main risk factors to reach strategies to control and reduce human and animal brucellosis.

**Results:** In the first phase, according to statistical analysis, 3363 persons including 47clusters were enrolled and considered for future studies. All participants were interviewed and demographic questioners were successfully completed. Finally, 2367 blood samples were entered in serology analysis. The seroprevalence of brucellosis based on serologic titers of Wright and 2ME test was 6.59% (95% CI: 5.62%: 7.66%) and 3.46 %( 95% CI: 2.72%: 4.20%) respectively.

**Conclusions:** In the first phase, an extensive range of data and information were collected as the basic data for the following phases of the cohort.

## Introduction

Global burden of human brucellosis ranges from 1 to 200 new cases per 10 million people per year.^1^ Humans usually infect by *Brucella spp.* as an incidental host through direct contact with infected tissues or blood of animals or by consumption of contaminated dairy.^2^ Brucellosis have various clinical manifestations, which often underdiagnosed and treated with delay. The majority of infected patients in endemic areas are asymptomatic and fewer than 10% of human cases with brucellosis may be recognized early after infection.^3, 4^ Clinical signs and positive laboratory finding are the basic methods to diagnosis of brucellosis. Isolation of the brucella from the patient’s blood, bone marrow or other tissue sample is the final diagnosis, but it is slow-growing and being intracellular are big restrictions to isolation.^5, 6^. Confirmation of a clinical diagnosis of brucellosis usually done by testing a sample of blood or bone marrow to detect Brucella bacteria or searching antibodies antigens the Brucella in blood samples. To sero-diagnosis of human brucellosis, different serological tests including the Rose Bengal plate test (RBPT), Serum agglutination test ^7^, 2-mercaptoethanol (2-ME), the plate agglutination test ^8^, Coombs and enzyme-linked immunosorbent assay (ELISA are available). In Iran, doctors usually use the Wright, Coombs Wright and 2ME tests because comparing to other new and expensive diagnostic tests, these tests are effective enough.

Like many developing countries, brucellosis in Iran is still one of the most puzzling problems for humans and animals health.^4, 9^ The aim of the current study as the first prospective cohort survey on brucellosis is to investigate more about different aspects of brucellosis including study clinical and sub-clinical forms of Brucella infection in a selected population, study the socio-demographic and behavioral risk factors and study the best way to true diagnosis the brucellosis in selected population during different phases.

At the first phase of this cohort study, we estimated the seroprevalence of brucellosis and epidemiological features and risk factors of the Brucella infection. The next phases of Famenin cohort of brucellosis is projected to start after finishing the first phase analysis and preparation of the arrangement for future studies and will last four years. The follow-up phases will be including more studies about A) Investigation and follow up of brucellosis disease in seropositive participants in Famenin cohort of brucellosis and their families using culture and serology methods. B) Evaluation of the incidence, relapse, clinical manifestations and complications of brucellosis during two years among participants. C) PCR Confirmation of results of a study the seroprevalence of brucellosis in Famenin brucellosis cohort. D) Determine the seroprevalence of brucellosis in the livestock population. B) Identification of human and livestock species, D) Determine antibiotic resistance. E) Study the Prevalence of microbial contamination of dairy products. F) Determine the pattern of disease transmission.

We hope to know more about different aspects of brucellosis and find the most important risk factors of brucellosis in the human and animal population in Fmenin to implement the best strategies to reduce the incidence and recurrence of brucellosis.

## Methods

### Selection of the participants

**Figure 1:**
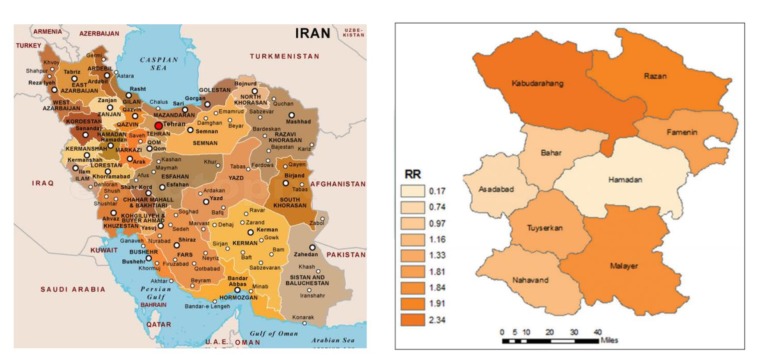
Geographic location of Hamadan province in Iran. B: The rate ratio of brucellosis in Hamadan province (2009–2015)

According to the report of the Ministry of Health and Medical Education in 2009, Hamadan province was a very high incidence region of brucellosis in Iran^10^. During 7 years (2009–2015), a total of 9318 cases of human brucellosis were registered in Hamadan province.^11^ Geographically Hamadan province is located in the west of Iran and has a population of 1758268(Figure 1). (As reported by Statistical Center of Iran’s census in 2011)

Hamadan province is divided into nine townships, which in turn are divided into 23 districts in total. These divisions are shown on the map as well as the neighbor provinces. Famenin is one of the Hamadan province's nine townships with a high incidence of brucellosis which located 55 km north east of the province. (Figure 1) It is 1628 meters above sea level and has mountainous climate. Famenin is considered as an important center of agricultural production in the province and almost of inhabits of Famenin are from the Turkic ethnic group mixed with the other native people.

In this study, due to the near distance and high incidence of brucellosis, Famenin was chosen as the target population for the setting of brucellosis investigation in Hamadan province. According to 2011 census, Famenin, s population was 43044. The number of population in urban and rural areas was 14478 and 28007 people, respectively.

The number of samples was calculated using the following formula.

$$n=\frac{z_{1-\frac{\alpha }{2}}^{2}(p)(1-p)}{{\left(d\right)}^{2}}$$

The incidence of brucellosis in 2015 in Famenin was 109 per 100,000 populations.^11^ Based on the fact that the instantaneous outbreak in the community is not available and according to the relationship between incidence and prevalence rates of brucellosis and considering the expert opinion, the prevalence of instantaneous outbreak was assumed equal or two times of instantaneous occurrence; Which in this formula was 0.00275 and the type one error was 5%. Considering the absolute errors of 0.002 and using the above formula, 2634 samples were calculated. By applying the effect of the sampling plan of 1.2, the required sample size was calculated 3161. The cluster sampling method was used which in it, after classification of Famenin population based on urban and rural areas, the clusters were selected systematically of each one. Each cluster included 10 households or 40 people. In the city of Famenin, with a population of 43044 people, the total number of samples was 3161 people including 1282 people from urban areas and 1879 people from rural areas. Of the 3161 selected people, 1,282 in 32 clusters were of urban population and 1879 people in 49 clusters were of rural population. To select the head of the clusters, the first and last postal code of each division was first obtained from the post office. Based on the aggregation of the postal codes, the number of clusters was proportional with the density of the postal codes of each division. Large divisions with more households accounted for more sample sizes. Then, using the systematic sampling, the postal codes of head clusters from rural and urban areas were selected. By using this systematic approach, we have provided the better and geographically representative distribution of eligible clusters. The address of the head of clusters got from the post office to dispatch the questioner and collect data. To select a random number to start systematic sampling, Excel software was used. All subjects of urban population and the villages of Famenin entered the study between Septembers to November 2016. Age less than two years old was not included in the study (Exclusion criteria). Based on cluster sampling, households in Famenin were determined by coding the areas and households.

### Data collection and experimental methods

In this study, trained interviewers (health care workers in the area) approached members of the targeted households and completed a Researcher developed questionnaire which includes data of sex, education, residency, occupation, history of Brucellosis, contact livestock history, using local dairy and kind of dairy products; and the next, handed them written invites to have a blood sampling for further analysis and completion brucellosis examination at the specified health centers. Also for every established case of brucellosis, a questionnaire containing five sectors and 72 questions was done for registration qualitative recurrence and complications of brucellosis as one of Brucellosis outcomes. At the first sector, demographic and general data like the step one was recorded and the next section was including laboratory and para-clinical symptoms diagnosis. Expert field workers checked twice and entered the collected data by a software program established in the brucellosis research center.

**Figure 2:**
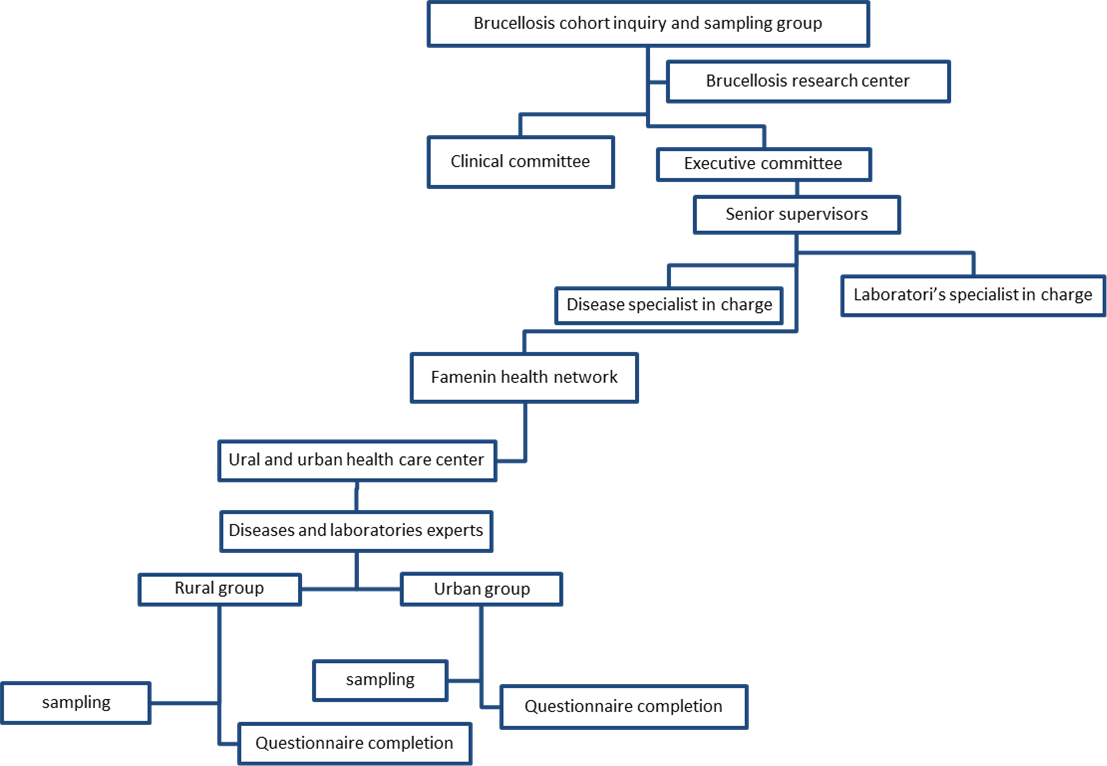
Regulatory flowchart of Famenin brucellosis cohort

All related workers were expert to identify symptoms of brucellosis. Written consent was obtained from all participants. All procedures from concept to completion observed regularly by the project’s supervisors. Figure 2 shows the Regulatory flowchart of Famenin brucellosis cohort. This study was approved by the Ethics Committee in Hamadan University of Medical Sciences (NO. 16.35.9.289) and written consent was received from all participants.

Next to obtaining the informed consent, all identified household’s people who referred to specified health centers first clinically visited and then blood sampling was done, and subjects were joined and follow-up was initiated. Blood samples have sent to integrated research laboratories at Hamadan University of Medical Sciences for further analysis.

Our field workers collected 5cc blood samples for serological analysis from each subject. They were expert in standard techniques for gathering and carrying blood samples. All the blood samples transported with cold boxes to the integrated research laboratories in Hamadan University of Medical Sciences in within 2-3 hours of sample collection. After isolation of serums, they were re-centrifuged again, were frozen and received special codes for future Brucella antibodies investigation and analysis. At the next step, taken blood samples were examined using Wright kits and 2-Mercaptoethanol (2ME) test made by Pasteur Institute of Iran for serological examination of brucellosis. All the people who complain of symptoms and signs of brucellosis were referred for more examination by specialists in infectious or collaborator physician in the fellow hospital in Hamadan, or clinic and health centers in Famenin city. Also, people who have positive serology and clinical symptoms will be referred to collaborator physicians for the treatment and after recovery and end of treatment, they will be followed up every 3 months for two years. People who have negative serology followed up again six months later. Phone calls to each eligible permitted quick announcement between trained workers and study control center for direct advice and to summon the medical doctor to assess patients with complicated brucellosis. At the end of the first phase of data collection, field workers made a final household checking and support participants with a leaving study if wanted. Our colleagues also controlled any missed data regularly. The first phase of the study was completed in the early of 2018.

Also, we will record new cases (annual incidence of disease) and population dynamics of affected and at the high-risk population (Veterinarians, farmers, butchers and slaughterhouse workers) based on recurrence and complications of the brucellosis within 4 years.

All gathered data were entered into SPSS software version 16 and analyzed using descriptive statistics, chi-square statistical tests and logistic regression model. The prevalence of brucellosis in different subgroups with a 95% confidence interval was reported. The significance level in all statistical tests was less than 0.05.

## Results

At the first phase of this study, an extensive range of data and information were collected as the basic data for the following phases of the cohort. At the end of sampling period of the first phase of this study, 3363 people were enrolled and interviewed which of them 2367 blood samples were gathered for further analysis; 67.8% from rural areas and 32.2% from urban areas; 44.8% 44.8% of which were male and 55.2% were female. (Participation rate of 70/38% included 70/5% urban participation and 87% rural participation). This variance was due to non-cooperation of some inhabitants for blood sampling and additional steps. From 2367 people, 1472 (62.1%) were married and 895 (37.8%) were single. Table 1 listed some of the more important measured aspects collected during the first phase of the cohort.

**Table 1:** Baseline characteristics of study participants

**Variables**	**Number**	**Percent**
Sex		
Male	1060	44.8
Female	1307	55.2
Education		
Illiterate	466	21.2
Elementary	916	41.2
Guidance	399	18.2
High School	145	6.6
Diploma	194	8.8
Super-Diploma And Higher	78	3.5
Residency		
City	761	32.2
Village	1606	67.8
Occupation		
Animal Husbandry	171	7.7
Farmer	258	11.6
Veterinarian	0	0.0
Vaccinator	1	0.1
Slaughterhouse Staff	1	0.1
Butcher	2	0.1
Lab Staff	0	0.0
Housewife	1006	45.8
Dairy Sales	2	0.1
Chef	2	0.1
Other Student and so on	879	40.1
History of brucellosis		
No	2182	92.6
Once	174	7.3
>once	1	0.1
Contact Livestock History		
Yes	785	33.2
No	1579	86.8
Using Local Dairy		
Yes	2082	88.4
No	274	11.6
Kind of dairy products		
Milk	1977	95.5
Skim	326	15.7
Cheese	644	31.0
Butter	237	91.6
Cream	200	17.6
Curd	366	17.9
Colostrum	479	2.3
Traditional Ice Cream	170	8.2

The seroprevalence of brucellosis based on serologic titers of Wright and 2ME test was 6.59% (95% CI: 5.62%: 7.66%) and 3.46 %( 95% CI: 2.72%: 4.20%) respectively. (Table 2) These basic data will be considered for future studies. The mean age of 2367 individuals was 34.57 (with a standard deviation of 9.20) who range from 2 to 95 years old.

**Table 2:** Frequency distribution of subjects according to the Wright test and 2ME test titers in Famenin

**2ME**	**Wright**						**Total**
	**1/80**	**1/160**	**1/320**	**1/640**	**1/2560**	**1/5120**	
Negative	61	12	1	0	0	0	74
1/20	17	7	0	0	0	0	24
1/40	12	6	1	0	0	0	19
1/80	10	7	6	1	0	0	24
1/160	0	5	4	0	0	0	9
1/120	0	0	0	1	1	0	2
1/640	0	0	0	3	0	1	4
**Total**	100	37	12	5	1	1	156

In addition, there was a significant difference between the mean age of subjects (42.15 ±19.8 years) with positive serology of Wright test and those (mean age: 34.03 ±20.88 years) with negative serology (*P*<0.001). Using logistic regression analysis, the odds ratio history of brucellosis and disease was 3.74 (2.818 to 4.963), (*P*<0.001). It means that the history of brucellosis has a positive effect on Wright test results. All basic data gathered in this step were documented and analyzed accurately for use in future studies.

## Discussion

At the start of this cohort study, from September to December 2016, from 3363 eligible ones, according to the sampling method, the questionnaire was completed. All participants were interviewed and questioners were effectively done.

In this study the main exposures are contact to domestic animal, consumption of unpasteurized dairy products, unsafe work in high risk groups (veterinarians, butchers, slaughter house staff and animal owners; The Outcomes are infected people with Brucella or brucellosis disease; The measurements interval is visiting the persons every 3 months and clinical examination looking for sign and symptoms of brucellosis and measurement methods are demonstrate rising titre of antibody to Brucella antigens in blood sample by Wright and 2ME tests.

Of our main strengths is that Famenin cohort of brucellosis is a big public cohort study approximately demonstrative of the Hamadan province’s population. An important strength is the presence of large population (comparing to just small population samples as studied earlier in Iran) which lets us to explore brucellosis infections between thousands of people. Other of our strengths are greatly dynamic methods of investigation for brucellosis exploiting were used including a range of face to face or telephone follows or text messages. Strong descriptions of brucellosis were based on a range of diagnostic methods including clinical symptoms and serology allowing the existence of maximum infection to be tracked. This study delivers us a wide range of information about social, behavioral and natural features concerning brucellosis spread, allowing examination of many research questions for next studies. Since brucellosis happens more in rural communities due to contact with infected animals and dairy products, a big part of our study were including people living in rural societies other than urban and developed area; therefore, they have features like to true disposed population, and our finding would be useful for those locations.

Famenin have a relatively stable population structure, and immigration frequency is very low there; so the results are valuable for review and follow-up at the future. This cohort study is the only prospective cohort which is population-based for investigation on brucellosis in Iran. Another advantage is the excessive volume of gathered information which will be helpful to study the brucellosis epidemiology and the great chance of investigate many other different aspects of brucellosis. To ensuring about the quality of the gathered information in this cohort, several methods have been used at the first phase in collecting basic data.

Due to delays in obtaining funding and some instruments and material for diagnosis and miss of cooperation of some people for blood sampling or other steps of study, results reported with a delay in the first phase of recruitment.

## Conclusion

Regarding our finding in different studies in Famenin cohort of brucellosis, we hope to reach some good preventive possible of executive and intervention programs to make evidence to reduce, control and prevent of brucellosis in human and animal populations. At this time, we encourage regularly program of animal vaccination in animal populations, pasteurized dairy consumer, education programs in rural and urban population and preventive education programs in at high risk groups.

## Acknowledgements

We thankful all 3363 participants who contributed our achievement in Famenin Cohort study. Also we want to appreciate all the staff members of Famenin health center and the staff of core facility of Hamadan University of Medical Sciences and anyone for their supports of this cohort study.

## Conflict of interest

Authors have no conflicts of interests to declare.

## Funding

The study was funded by Vice-chancellor for Research and Technology, Hamadan University of Medical Sciences (Grant No. 9504222003). This granting body has not been assigned for publication fee.

## Highlights

- This study is the first prospective cohort survey on brucellosis in Iran.
- The main goal in this cohort study is to know more about different aspects of brucellosis and find the most important risk factors of brucellosis in human and animal population.
- The extra information collected at the first phase of this study is described for the first in Iran.
- We hope to reach the preventive and controlling methods to decline the brucellosis by following studies in Famenin brucellosis cohort during next years.

## References

[1] Buzgan T, Karahocagil MK, Irmak H, Baran AI, Karsen H, Evirgen O (2010). Clinical manifestations and complications in 1028 cases of brucellosis: a retrospective evaluation and review of the literature. Int J Infect Dis.

[2] de Figueiredo P, Ficht TA, Rice-Ficht A, Rossetti CA, Adams LG (2015). Pathogenesis and Immunobiology of Brucellosis: Review of Brucella–Host Interactions. Am J Pathol.

[3] Salari M, Khalili M, Hassanpour G (2003). Selected epidemiological features of human brucellosis in Yazd, Islamic Republic of Iran. East Mediterr Health J.

[4] Afsharpaiman S, Mamishi S (2008). Brucellosis: review of clinical and laboratory features and therapeutic regimens in 44 children. Acta Med Iran.

[5] Acharya KP, Kaphle K, Shrestha K, Bastuji BG, Smits HL (2016). Review of brucellosis in Nepal. Int J Vet Sci Med.

[6] Alavi SM, Mugahi S, Nashibi R, Gharkholu S (2014). Brucellosis risk factors in the southwestern province of Khuzestan, Iran. Int J Entric Pathog.

[7] Poorhajibagher M, Pagheh A, Nasrollahi M, Mesgarian F, Badiee F, Ajami A (2012). The evaluation of seroprevalence of brucellosis in patients refering to health care center of Gonbad Kavoos, 2009-11. Journal of Mazandaran University of Medical Sciences.

[8] Christopher S, Umapathy B, Ravikumar K (2010). Brucellosis: review on the recent trends in pathogenicity and laboratory diagnosis. J Lab Physicians.

[9] Bokaie S, Sharifi L, Alizadeh H (2008). Epidemiological survey of brucellosis in human and animals in Birjand, east of Iran. J Anim Vet Adv.

[10] Khazaei S, Shojaeian M, Zamani R, Mansori K, Mohammadian-Hafshejani A, Rezaeian-Langroodi R (2016). Epidemiology and Risk Factors of Childhood Brucellosis in West of Iran. International Journal of Pediatrics.

[11] Khazaei S, Karami M, Mohammadbeigi A, Ayubi E, Shojaeian M, Mansouri K (2018). Spatio-Temporal analysis of brucellosis in Hamadan Province, West of Iran: 2009–2015. Advances in Human Biology.

